# Update on COVID-19 and Effectiveness of a Vaccination Campaign in a Global Context

**DOI:** 10.3390/ijerph191710712

**Published:** 2022-08-28

**Authors:** Ioannis Alexandros Charitos, Andrea Ballini, Roberto Lovero, Francesca Castellaneta, Marica Colella, Salvatore Scacco, Stefania Cantore, Roberto Arrigoni, Filiberto Mastrangelo, Mario Dioguardi

**Affiliations:** 1 Department of Emergency and Urgency, National Poisoning Center, Riuniti University Hospital of Foggia, 71122 Foggia, Italy; 2 Department of Precision Medicine, University of Campania “Luigi Vanvitelli”, 80138 Naples, Italy; 3 AOU Policlinico Consorziale di Bari-Ospedale Giovanni XXIII, Clinical Pathology Unit, Policlinico University Hospital of Bari, 70124 Bari, Italy; 4 Interdisciplinary Department of Medicine, Section of Microbiology and Virology, School of Medicine, University of Bari “Aldo Moro”, 70124 Bari, Italy; 5 Department of Basic Medical Sciences, Neurosciences and Sense Organs, University of Bari “Aldo Moro”, Piazza G. Cesare 11, 70124 Bari, Italy; 6 Independent Researcher, Sorriso & Benessere-Ricerca e Clinica, 70129 Bari, Italy; 7 CNR Institute of Biomembranes, Bioenergetics and Molecular Biotechnologies (IBIOM), 70125 Bari, Italy; 8 Department of Clinical and Experimental Medicine, Università degli Studi di Foggia, 71122 Foggia, Italy

**Keywords:** SARS-CoV-2, clinical microbiology, human vaccines, COVID-19, pandemic, global health

## Abstract

The COVID-19 pandemic caused by SARS-CoV-2 remains a significant issue for global health, the economy, and society. When SARS-CoV-2 began to spread, the most recent serious infectious disease of this century around the world, with its high morbidity and mortality rates, it is understandable why such infections have generally been spread in the past, mainly from international travel movements. This perspective review aimed to provide an update for clinicians on the recent developments related to the microbiological perspectives in pandemics, diagnostics, prevention (such as the spread of a virus), vaccination campaigns, treatment options, and health consequences for COVID-19 based on the current literature. In this way, the authors attempt to raise awareness on the transversal nature of these challenges by identifying the main risk/vulnerability factors that the scientific community must face including our current knowledge on the virus capacity of the mechanism of entry into the cells, the current classifications of viral variants, the knowledge of the mathematical model on the spread of viruses (the possible routes of transmission), and the effectiveness of vaccination campaigns in a global context of pandemic, particularly from COVID-19, with a look at new or future vaccines.

## 1. Introduction

Microbiology is a branch of medicine and biology that studies the structure and functions of microorganisms (i.e., all those single-celled, multicellular or acellular living organisms not visible to the naked eye such as bacteria, Archaea, some types of fungi, algae, protozoa, viruses and prions) [1]. Compared to bacteria, viruses do not have organelles, nuclei, or even ribosomes, and the replication mechanism depends exclusively on the viral genome. In particular, DNA viruses usually use host cell proteins and enzymes to make additional DNA, which is used to copy the genome or be transcribed to messenger RNA (mRNA), which is then used in protein synthesis. RNA viruses such as the influenza virus usually use the RNA core as a template for the synthesis of viral genomic RNA and mRNA. The viral mRNA is translated into viral enzymes and capsid proteins to assemble new virions [1,2].

Current technical–scientific knowledge in the biomedical field is used to study the virulence of pathogenic microorganisms already naturally present in the environment, and is the first point of analysis for examining how these pathogens become devoid of pathogenetic capacity. There may be many characteristics that make a microorganism difficult to treat in the field of health management [2] such as:

1. Insensitivity to traditional vaccines and normal immunological factors.
2. Drug (antibiotics and others) resistance.
3. Their persistence in the natural environment despite the action of disinfectants.
4. The production in large quantities of already known or “new” biotoxins.

**Figure 1:**
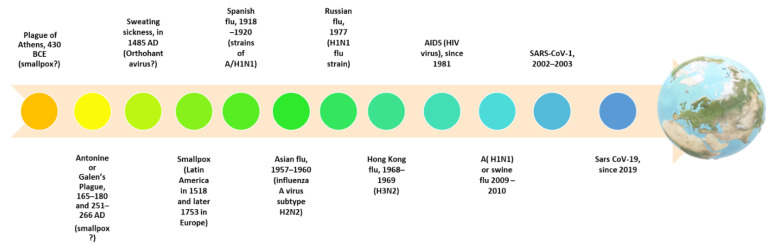
The timeline of pandemics due to viruses. Identifying a causative agent in antiquity is difficult, as in the case of the Antonine plague, which follows the descriptions of Galen; the infection was most likely caused by smallpox or measles.

It is worth underlining that the pathogens represented by microorganisms such as viruses show their effects after several days, and are responsible for epidemics or pandemics being more dangerous due to greater opportunities for transmission, especially inhalation. Indeed, since ancient times, we have descriptions of epidemic or pandemic conditions that have plagued humanity (Figure 1) [3,4,5].

According to the World Health Organization (WHO), there are three conditions for a pandemic to occur: (a) the appearance of a new pathogen for which effective treatments are not known; (b) the ability of this agent to affect human beings; and (c) the ability of this agent to spread rapidly through contagion. Other factors can be environmental, ecological, and sociological influences that affect the likelihood of pathogens encountering new hosts. In fact, many of these factors alter the spread of pathogens and their hosts or vectors [6].

Most of the viruses that have caused pandemics are zoonotic (i.e., they originate from interspecies contagion). These include the influenza virus, SARS-CoV-1, and, as far as we know, COVID-19. Finally, in emergency public health situations such as epidemics or pandemics, contagion is more frequently associated with RNA viruses. This is probably due to the greater genomic flexibility these viruses [6,7].

## 2. The New Challenge of SARS-CoV-2

COVID-19 is a disease that spread rapidly around the world in 2020. It manifests both asymptomatically and symptomatically and primarily affects the respiratory and vascular systems. As of 5 December 2021, nearly 265 million confirmed cases, with over 5.2 million cumulative deaths, have been recorded, making it the deadliest pandemic since the Spanish flu [8]. Coronaviruses (CoVs), which are RNA viruses, belong to the *Coronaviridae* family (genus *Betacoronavirus*, subgenus *Sarbecovirus*). Some positively polarized single-stranded RNA viruses have a large outer envelope and multiply in the cytoplasm of animal cells that act as receptors. Most coronaviruses (CoVs) belonging to the *Sarbecovirus* subgenus are found in bats [7]. The main differences between this new SARS-CoV-2 virus and SARS-CoV-1 are found in the former’s spike protein as well as in glycoprotein S, which determines the specificity for the epithelial cells in the respiratory tract. In fact, it can bind the ACE2 receptor (angiotensin 2 converting enzyme), expressed by the capillary cells of the lungs, and has a greater affinity for ACE2 than SARS-CoV-1. Both viral replication and the viral spike protein alone have been shown to selectively reduce the expression of ACE2. ACE2 hydrolyzes the Ang I, generates Ang- (1–9) and the Ang II generating Ang- (1–7), which binds to Mas (a receptor endogenous, coupled to phospholipase C, and is expressed in various organs such as the heart, vessels, testes, and brain) to play its main role, that is, antagonizing many of the effects mediated by Ang II. The Ang- (1–7) counterbalances the effects of Ang II and its produced activity of ACE2. In the lungs, the activity of RAS, ACE, and Ang I are intrinsically high, but also the activity of ACE2 is very high, and this is to regulate the Ang II/Ang- (1–7). In practice, this is to limit the effect of Ang II, because high levels of Ang II in the lung can lead to increases in vascular permeability and edema. In addition, SARS-CoV-2 induces a rapid downregulation of ACE2 from the cell surface and the release of active catalytic ACE2 ectodomains. Thus, the physiological balance between ACE/ACE2 and Ang II/Ang- (1–7) is likely to be disrupted by SARS-CoV-2 viral infection [7].

**Figure 2:**
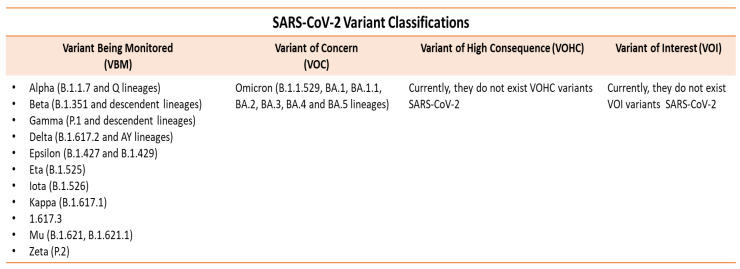
The classification of the SARS-Cov-2 variants. The World Health Organization (WHO) classifies the variants in four categories. According to the WHO, the variants being monitored (VBM) is the general term for all variants of concern and variants of interest that are not the Delta variant. A VOC variant must have one or more of the following characteristics: it must have demonstrated that it increases the severity of the disease, the contagiousness, the treatment, or the vaccine has a reduced efficacy. According to the WHO, other variants are the VOI (variant of interest) and VOHC (high-risk variant), but neither is designated for SARS-CoV-2. Some interesting variants of the VOC are the English (Alfa, B.1.1.7), which has been shown to have 37%> transmissibility compared to non-variant strains with great statistical uncertainty between 18% and 60%; the African (Beta, B.1.351) identified in South Africa; and the Brazilian (Gamma, P.1), which demonstrated a potential greater transmissibility and a possible risk of reinfection, but there is no evidence on the greater severity of the disease. The designed VBM are the Indian (Delta, B.1.617.2) and AY lineages and in South Africa, the Omicron (B.1.1.529) on 24 November 2021. Further research is underway to better understand the impact of mutations on the virus behavior and to ensure that all appropriate public health interventions are taken. Source: Centers for Disease Control and Prevention, (CDC), USA. (Available from https://www.cdc.gov/coronavirus/2019-ncov/variants/variant-classifications.html#anchor_1632158885160, accessed on 29 July 2022) [10].

The organization of the SARS-CoV-2 genome has been detected by PCR in many biological samples derived from, for example, the lung tissue, kidney, sputum, throat smear, upper airway aspiration, and flushing fluids [7]. The virus is thus investigated by isolating it in cell culture, detecting it with an electron microscope, and demonstrating specific gene sequences by polymerase chain reaction (PCR) and the “microarray” method as well as the detection of antibodies by indirect immunofluorescence tests [8]. The virus can have (a) a mutation that is a single change in the genetic code of a virus; (b) a variant that means that the viral genetic code can contain one or more mutations; and (c) a lineage that creates a group of viruses that are closely related to a common ancestor (SARS-CoV-2 has many lineages and all cause the same infectious disease). In fact, it presents mutation, recombination, and reassortment phenomena that allow it to overcome species barriers, adapt to different new hosts, and give rise to new variants that may be designated by public health organizations as variants of concern (VOC) or variants of interest (VOI) that may require public health intervention (Figure 2) [7,9,10].

## 3. Current Knowledge about the Spread of Viruses

In general, the interactions between the virus and host can therefore be divided into four types: (a) *stable*, keeps the virus in the environment (the host and viral populations are in equilibrium and are maintained in the environment), characteristic behavior of the virus in the primary host or reservoir; (b) *in evolution*, so the passage of the virus from the traditional population to a new one, belonging to the same host species or to different species, with progressive adaptation of the pathogen; (c) *dead-end host*, that is, the passage of the virus from the traditional host to a new one in which the virus is unable to transmit effectively for which it does not adapt, so may also be due to the high lethality of the virus which, causing the too rapid death of the new host, does not allow its transmission; and (d) *resistant,* so the infected host completely blocks the replication cycle of the virus (non-receptive host) [1,7]. To infect a new host, a virus must be able to overcome the species barriers and carry out its replication cycle in its cells; this process can be limited at various levels including binding to the receptor, entry into the cell, the replication, gene expression, and the spread of an infecting progeny. The use of an effective transmission route and the avoidance of the immune response are also important factors. A leading role is certainly played by the ability to bind a specific cellular receptor; to have the species jump of a virus, the potential new host must have a receptor recognized by the virus on the cell surface so that it can bind to it and then enter the cell. We can categorize viruses into two groups: those that are able to recognize and bind to various cell receptors, and that are therefore more predisposed to overcoming species barriers and infect different hosts, and those that are able to recognize and bind to only one type of cell receptor, and that therefore have more difficulty in overcoming species barriers. Similarly, viruses that bind to various cell receptors between different animal species are more able to jump between species, while those that bind to much more specific receptors find this more difficult as they have to undergo greater evolution (i.e., adapt); in most cases, emerging viruses do not immediately have a good capacity for adaptation to a new host and are therefore only able to transmit for a few replicative cycles [7,11,12]. Therefore, the main obstacle faced by the appearance of a new virus following a species jump is represented by adequate viral transmission between different individuals, from the infected to the healthy, thus creating a new host species. From this, we understand that the possibility of a pathogenic virus causing a disease depends on the interaction of many factors derived from both the microorganism and the vector [12,13].

As with other infectious diseases, the amount of viral load (i.e., the number of microorganisms transmitted from one person to another) is probably the main cause of infection. The magnitude of the viral load is determined by: (a) the secretions of the patient who is the source of the infection, and (b) the distance between the original patient and the person to whom it is transmitted (places with a large crowd, for example). The infection therefore varies over time, even during the symptomatic phase of the disease, and the virus is transmitted more easily in the advanced stages of the disease [13,14].

One of the models for obtaining demographic and epidemiological data is the SIR. The term of the SIR model is an acronym, where “S” stands for *Susceptible* (i.e., the healthy individuals who can contract the disease), “I” stands for *Infectious* and are the individuals who have contracted the disease and are able to pass it on, and finally “R” stands for *Recovered or Removed* (i.e., individuals who are cured), and for SIR, they cannot be part of the transmission process (i.e., the disease determines a permanent immunity). Thus, regarding the concept of infectious being when a pathogen establishes itself in an exposed individual, they become infected. Infected individuals who can transmit the disease are called infectious. In general, infected individuals may not be infectious for a so-called latency period. Historically, the use of deterministic and probabilistic models in the analysis of the development of infectious diseases has spread widely since 1760, when developed the first mathematical model was formulated by D. Bernoulli to support vaccination against smallpox. This was followed by Hamer in 1906 and Ross in 1911, which were aimed at the perception of malaria, and in 1927, the SIR model by Kermack and McKendrick was developed to explain the rapid growth and subsequent decrease in the number of infected people observed to some degree in that epidemic’s era such as the plague or cholera. Subsequently, from the second half of the twentieth century, there was an increasing number of models and mathematical studies that were not only deterministic models, but some could also consider the stochastic effects [15,16]. The SIR provides the simplest representation of the spread of an infectious disease that is transmitted by contact [17]. In this model, we introduce the total birth rate Λ and the total death rate per person μ, both assumed to be constant. Everyone born is assumed to be susceptible, while μS, μI, and μR represent the total death rate in the susceptible, infected, and repressed class, respectively. Thus, we can write the SIR model with the demographics in this two dimensional system:

S′(t) = Λ − βIS − μS, I′(t) = βIS − αI − μ I

**Figure 3:**
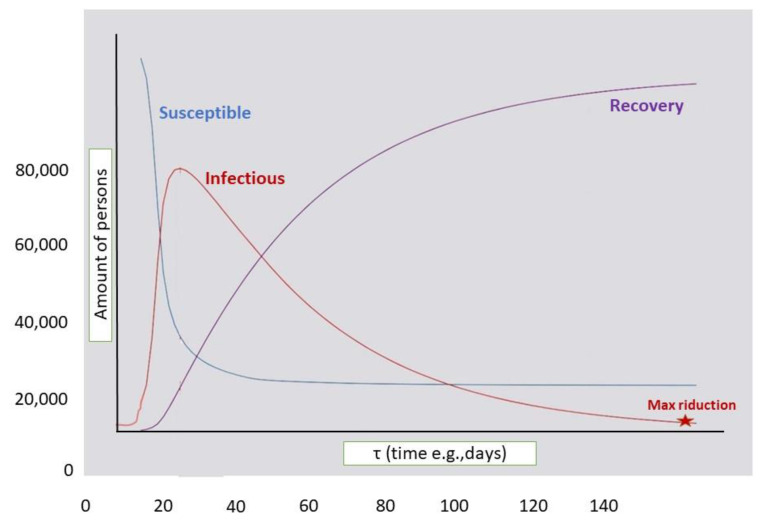
Example of the three curves in relation to the time/quantity (number of people) from daily data modeled with the epidemiological procedure SIR that divides the population based on three consecutive stages: susceptible, infected and removed (equal to the sum of healed, immune, and deceased).

Thus R′ = N − S − I

where α is the probability that a single infected individual heals in the unit of time, and β is the constant transmission rate while the product I, a function of time, is called the infection force [16,18]. Therefore, the modality that leads to a viral emergency requires subsequent adaptations to the new host before developing adequate transmissibility. In fact, to evaluate this possibility (i.e., the emergence of a pathogenic microorganism), the basic reproductive number (R0) was created. The parameter R0 is called the basic reproductive number of the disease and is given by the ratio R0 = βΛ/µ (α + µ) (Λ is measured in the number of people per unit of time; α is the probability that a single infected individual will heal in the unit of time; β is a constant transmission rate; μ is the total death rate per person) (Figure 3) [16,17,18].

In Wuhan, during the lockdown, the infected reached their maximum about 35 days from the start, and the latest infection was observed 70 days from the start. In total, the population involved in this phase of the epidemic was about 80,000 people out of about 10,000,000. These 80,000 people were tracked and isolated in an extremely rigorous way, managing to extinguish the epidemic in just over 2 months after the start. Epidemiologically, the basic reproductive number provides the number of secondary cases that a single infectious individual produces in a population of all susceptible individuals. In fact, if the whole population is susceptible at the time of the arrival of the “index case”, it means that it will find several of the susceptible equal to the carrying capacity, which is the ratio Λ/μ. Instead, the sum α + μ is the rate with which an individual “leaves” (it is no longer infected) the population of the infected and therefore the average time that each individual spends as infectious is 1/α + μ unit of time [16]. The number of transmissions per unit of time is given by the βSI incidence (S and I are measured in number of people). If it is assumed that there is only one infectious individual, (I = 1), while the rest of the population is susceptible, S = Λ/μ, then the number of transmissions per unit of time made by that single individual is βΛ/μ. Thus, the number of transmissions that a single individual can make during as long as they are infectious will be βΛ/μ (α + µ), which is precisely the expression of R0. Then, the number of newly infected individuals generated by an already infected individual in each fully sensitive population is assessed, depending on the number of infected-healthy contacts, the probability of transmission, and the infectiousness of the pathogen. Thus, the R0 number represents the calculation of the degree of adaptation of a given pathogen to infect each host effectively. Therefore, if R0 shows values <1, the infection will limit itself and the pathogen will start disappearing (because it will not be able to stay in the population) [15,16,17]. On the other hand, if R0 is >1, the infection will spread in the population and the pathogen will stabilize in it. In fact, if the value of R0 is close to 1, the infection will have an epidemic character, while if the value is higher than 1, the infection will have an endemic character R0 >1. In the case of a virus, it will be difficult because as soon as the species jump has been made, it will show an R0 value greater than or equal to 1; however, the evolutionary adaptation that the pathogen can begin follows this event, while factors related to the host and the environment can lead to an increase in the R0 value and the stabilization of the infection in the new host. Therefore, the possibility of the virus mutating comprehensively influences its ability to acquire a value of R0 >1 and spread [19]. To mathematically understand the meaning of the basic reproductive number as we mentioned before, it is necessary to obtain the solutions of the two dimensional system belonging to the SIR:

x′ = ρ (1 − χ) − R0χy, y′ = (R0χ − 1)y

Thus ρ = μ/α + μ and R0 = βΛ/μ (α + μ)

where ρ and R0 they are both dimensionless parameters [16]. Thus, the probability that the adaptation of the virus to the new host takes place successfully, and that therefore the pathogen remains in it, depends on four factors: (a) the number of primary infections, determined by the amount of virus that is transmitted to the new host, by the number of infected individuals, and by the repetition of more transmissions between the primary and new host; (b) the value of R0 in the new host at the beginning of the transmission, where the higher it will be, the greater the probabilities; (c) the number of mutations of genetic changes necessary for the virus to adapt and to complete its replication cycle in the new host; and (d) the characteristics of the pathogen (i.e., the genetic variability of the virus), the probability that the necessary mutations will occur, and how much R0 will change with each mutation. The genetic and evolutionary mechanisms that determine the jump of viral species, that is, the transmission of a virus from a traditional host to one previously resistant to infection, are still partially unknown [20,21]. For example, supposing that in the event of an epidemic in a school, some pupils found with symptoms were immediately confined to an isolated place (e.g., in the infirmary), with the report also indicating that only 1 in 130 people showed symptoms. Therefore, it is reasonable to assume that transmission in any case ceased once there was confinement to the school infirmary. Assuming the isolation in the infirmary occurred within 24 h of the onset of symptoms, then how does this affect the R0 estimate for 12 h? The formula for estimating R0 is R0 = β_1_ /γ + 1. If the pupils have been placed in isolation, the realized removal rate is greater than the natural recovery rate. Since this rate is in the denominator of the estimation equation, using the natural recovery rate positively skews our estimate because it is too large. For the 24 h isolation, we have γ = 1, which yields R0 = 1.094913 + 1 ≈ 2.09. For isolation in 12 h, we have an infectious period of γ −1 = 0.5, which implies γ = 2, yielding R0 = 1.094913/2 + 1 ≈ 1.55 [22].

**Figure 4:**
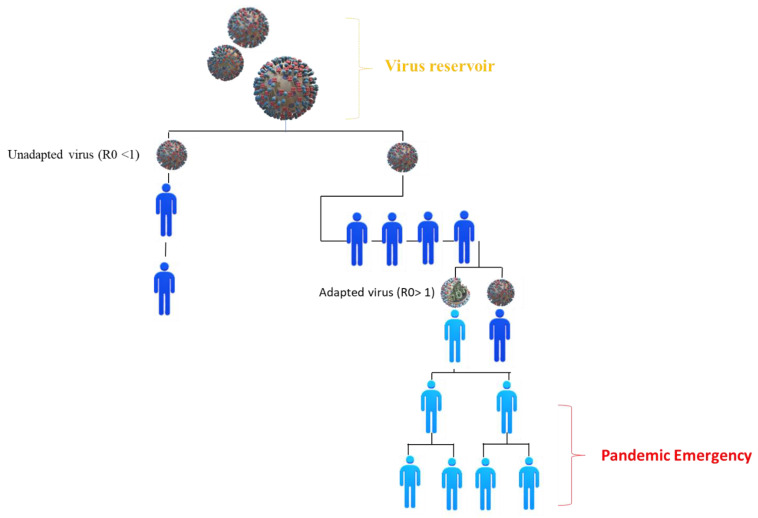
One of the possibilities of the transmission modalities in the new population between non-adapted viruses with R0 < 1 (the virus does not transmit further) and adapted with R0 > 1. R0 expresses how many people a case can infect at the beginning of the epidemic, that is, when there is no immunity in the population and interventions to limit transmission have not started. If, for example, R0 is equal to 3, each case can infect three other people on average, and these in turn another three each, and so on. As a result, the number of cases gradually increases, and a widespread dispersion ensues. If R0 is less than 1, there is no risk of an epidemic. This is because, in this case, one case can infect another person and thus the transmission gradually decreases. The emergence of a non-pre-adapted virus in a new host follows four consecutive moments: (a) exposure in which it is the contact between the donor host and the recipient (it can be influenced by geographical, ecological and behavioral factors); (b) the infection, in other words, the passage of the virus from the donor host to the recipient (which can be influenced by the ability of the virus to overcome the species barriers and by the compatibility with the new host, i.e., its binding to the cellular receptor, the ability to carry out the replicative cycle, evasion of the immune response and more); (c) diffusion such as the transmission of the virus between subjects belonging to the new population, which can be influenced by the ability of the virus to complete its replication cycle in the new host and from the contact between the subjects that make up the new population; and (d) the adaptation that is the evolution of the virus in the new host in order to remain in equilibrium within the population, which can be influenced by the genetic variability of the virus. Only the first two phases are essential to have the transmission of a pathogen in a new host, with the following two, there is the spread and stabilization of the pathogen in the new population. This difference makes it possible to distinguish the two types of transmission to a new host; there can also be dead-end host transmission with the infection of the new host (with or without disease), which, however, does not lead to the involvement of other subjects other than those directly infected by the primary host or, instead, the transmission with diffusion and adaptation of the pathogen in the population through the sick–healthy contagion between subjects belonging to the new host species (blue person: virus has not adapted with R0 < 1. Light blue person: virus has adapted with R0 > 1). Credits: Original figure by I.A. Charitos [7,15,16,23].

The prevention of future species leaps and the emergence of new pathologies, especially those of a zoonotic nature, requires, on one hand, the understanding in more detail of which mechanisms allow the virus to overcome the species barriers and adapt to a new host and, on the other hand, the identification and evaluation of the existing relationships between hosts that have a high potential to transmit new infections to other populations and hosts that show a high risk of acquiring new pathogens. Finally, an important distinction beyond the pandemic, which has a direct impact on the structure of the SIR mathematical model, is that between epidemic and endemic. As we know, an epidemic is an infectious disease that is extremely localized over time and its expansion is so rapid that the births and deaths of individuals are negligible. On the other hand, an endemic is an infectious disease that persists for a long time (albeit with varying levels of diffusion) and which, in its mathematical version, requires terms of birth and death. To make the analysis simpler, it was assumed that these new terms were in equilibrium to keep the total population “N” constant and that the offspring were all entered directly into the susceptible class “S”. Furthermore, the fewer the number of mutations required for the virus to achieve this goal, the greater the chances that this can occur (Figure 4) [15,16,19].

When the new coronavirus outbreak was recognized in China, one of the priorities was to estimate an important epidemiological parameter, which was the base reproduction number (R0). In the case of COVID-19, R0 shows that for each infection, 2–4 infections are directly generated, but the days of latency of infectivity vary from three to four. Thus, if we consider R = 4, the number of infectious cases will quadruple [7]. This assessment, coupled with the lack of immunity in the population, underlines that the novel coronavirus has the potential to lead to a widespread epidemic if measures are not taken, as has been found in practice in many countries. The protective measures that have been taken in order not to spread the virus among people are geared toward both individual and behavioral protection. These measures include avoid touching the face (eyes, nose and/or mouth), in other words, only touching the face with washed or decontaminated hands (with soap and water for at least 20 s or with solutions, gels, or alcohol-based tissues); physical distancing (one meter), which should be used with friends, family, and co-workers; the avoidance of social encounters and close contact with infected people; using the crook of the elbow or handkerchief when coughing or sneezing; wearing a face mask indoors and outdoors whenever physical distancing with other people cannot be guaranteed [24,25]; using medical face masks in these crowded, enclosed places, which reduces the risk of spreading the virus; and staying at home (self-isolation) when presenting with symptoms indicative of COVID-19 and calling for a general practitioner. Regarding travelers, in addition to the use of a mask and physical distancing (airports, railway stations, trains, ships, buses, or other), if they had symptoms such as fever, cough, or sore throat (or other symptoms of COVID-19), they must not travel, and if symptoms occur within 14 days of returning from travel, they should consult a doctor or primary care physician immediately [25]. When interventions are implemented to limit transmission such as social distancing measures, the focus shifts from R0 to the active reproduction number Rt. This indicator expresses the number of people an outbreak can infect in the presence of these interventions. Its value may change over time as the gradual introduction of measures and changing population behavior (e.g., hand hygiene, contact restriction) make transmission increasingly difficult. The goal is to reduce Rt to a level below 1, as this indicates that outbreak control has been achieved. In China, for example, both in the epicenter (Wuhan region) of the epidemic and in other regions, the lockdown brought the Rt very close to 0. The same happened in several countries in Europe with an Rt below 1. This estimation can be updated at regular intervals based on the data collected by epidemiological surveillance (cases diagnosed per day) by applying the appropriate methodology [26,27,28]. In this way, the course of the epidemic and the effectiveness of the measures can be assessed in real-time, if possible, as there is inevitably a delay from the moment a person is infected until diagnosis. It is important to note that even if the containment of the epidemic and the reduction of Rt to low levels have been achieved, the lifting of the measures may lead to an increase. Therefore, in the phase of phasing out, the monitoring of Rt is very important as it will allow for corrective actions to be taken if it is detected that it approaches or exceeds 1. The challenge is to find the combination of measures that allow for Rt to be kept below this value, obtaining at the same time the gradual recovery of economic activity, and more generally, the return of society to a pace that is as normal as possible [7,16,28]. For this reason, it is important to strictly follow the recommendations for hand hygiene, keep a physical distance, limit the number of people in enclosed spaces, etc. The monitoring of RT at the local level will allow for the identification of specific areas of greater dispersion and the implementation of targeted measures. A peculiarity of a new epidemic is that it is necessary to take measures without knowing in advance what their effectiveness will be [29], for example, we must go into lockdown or milder measures may be enough. In practice, it is not feasible to design a study in which different interventions are applied to individual populations to assess which are the most effective. Furthermore, a soft strategy that has proven effective in one country does not necessarily mean that if adopted elsewhere, it will have the same effect. The mathematical models make it possible to carry out a theoretical assessment incorporating hypotheses for various intervention scenarios (i.e., lockdown, closure of schools only, control and isolation of cases, etc.) and estimating for each of them the possible course of the epidemic [16,29].

## 4. Microbiological Perspectives in Pandemics

### 4.1. Diagnostic Tests and Screening

The rapid recognition of a microorganism such as this virus comes from collaborative research that uses high-tech laboratories with access to manifold techniques, from cell culture to electron microscopy and molecular microbiology. This demonstrates how a very well-organized effort may be able to respond to the threat of new infectious diseases that may arise in the 21st century [8,13,30]. Experience with SARS-CoV-2 also points out that a lack of cooperation can seriously hamper scientific progress and have serious consequences. It is not always known whether a microorganism such as SARS-CoV-2 can be permanently eliminated. It is up to researchers to rapidly detect and monitor pathogens for the health of humanity. Misdiagnosis of the etiology of an infectious disease could alter the trajectory of patient care, resulting in the unnecessary execution of diagnostic tests or prescriptions of antiviral, antibiotic, antifungal, and antiparasitic medications [30,31]. The abuse of antibiotics often reflects the difficulty of clinically discriminating bacteria from viral infections or other similar diseases. Thus, medical history, physical examination, and other ancillary examinations often do not provide definitive discrimination. To address this problem, effective tests must be put in place for the rapid recognition of bacterial or viral diseases [13]. In this field, microbiological research needs to be expanded for adjuvant therapy with bacterial strains that are beneficial to the human microbiota, and which use probiotics and stem cells during viral infection [32,33,34,35,36]. Moreover, because the aspiration of oral bacteria induces the expression of angiotensin-converting enzyme 2, a receptor for SARS-CoV-2, and production of inflammatory cytokines in the lower respiratory tract, poor oral hygiene can lead to COVID-19 aggravation [37,38,39,40,41].

The main resources for microbiology laboratories are antigen or antibody tests as well as pooling procedures or specimen collections. One of them is called immunoXpert ™, an ELISA-based test that accurately distinguishes between bacterial and viral infections and that measures the circulating levels of three host proteins that exhibit distinctive expression and complementary dynamics in host responses against bacterial and viral infections. These are as follows: (a) ligand-induced apoptosis related to tumor necrosis factor (TRAIL); (b) interferon-gamma inducible protein-10 (IP-10); and (c) C-reactive protein (PCR) [24]. The availability of diagnostic methods, in addition to allowing for the rapid diagnosis of SARS-CoV-2 infection, will allow us to better understand the period over which the viral load spreads (and the ability to transmit the infection) during recovery as well as to detect the presence of the virus in various biological fluids and its presence and excretion during the incubation period [7].

The throat swab searches for the progressive infection (as it searches for whether the virus is present in the airways at the time of examination), while the serological test searches for antibodies that the body has produced against the virus (it evaluates the immunological response produced). Serologic tests may not be as reliable in very recent infections compared to swabs, but they are a tool that allows one to establish whether the body has developed the related antibodies against COVID 19 (it measures the immune system’s response to infection) [42]. There are two types of serological test. The first is a rapid qualitative test (results within 15 min), which is measured through a drop of blood with a lancing device and evaluates whether the person has met the virus by searching for antibodies. The second is a quantitative test, which is exacted through venous sampling and detects the quantity of antibodies. Therefore, both tests detect the immunoglobulins IgM, IgA, and IgG [43]. In a case of acute infection, the presence of IgM is indicated (it is produced first). If the test detects IgG, it means that the infection occurred a while ago (the IgM decreases with the passage of time while the IgG that remains in the blood for life as an immunological memory begins to increase). The first immunoassay was carried out by the Wuhan Institute of Virology in 2020, and others have subsequently been described such as those using rapid detection with the GICA technique (colloidal gold immunochromatographic assay) [44].

Diagnostic tests are performed when a person has signs or symptoms of infection or when they are asymptomatic but have a recent history of known or suspected exposure. However, some diagnostic tests are licensed for use only in symptomatic individuals. In addition, a COVID-19 screening test searches for individual infections in a group (it involves asymptomatic individuals with no known or suspected exposure) to make individual decisions based on the test results [42].

Screening tests are different from diagnostic tests from a health benefit point of view. There are various social communities (such as schools, workplaces, health professionals, etc.) that establish programs for authorized screening tests of asymptomatic individuals with no known or suspected exposure; various testing options are used in these communities. A licensed test is used to perform a screening that is highly sensitive and has fast response times. If the screening test does not have these characteristics, the use of a less sensitive authorized point-of-care test (such as antigen detection) should be considered [43,44].

It is important to note that tests, even in a series, are of limited value if appropriate measures such as quarantine for those who are positive, social distancing, and mask use—even for those who are negative—are not also implemented. Negative outcomes should also be considered “supposedly negative” by health professionals and should be studied in the context of clinical and epidemiological information as well as patient history. If there is doubt with a negative antigen test, the result of a different test may be as different as the highly sensitive authorized molecular one.

Regardless of the test selected, it is important to monitor updates from national or international health control organizations such as the National Institutes of Health and Centers of Disease Control and Prevention (CDC), the FDA, and the WHO as well as the test developer for new information regarding the performance of the selected test with emerging virus mutations in the community [44,45]. It is important to note that tests, even in a series, are of limited value if not combined with appropriate mitigations for individuals who test positive such as quarantine, good contact traceability, effective behavioral protocols (such as wearing a mask), handwashing, and social distancing, even for subjects who test negative [45,46].

### 4.2. Vaccination Campaign

**Figure 5:**
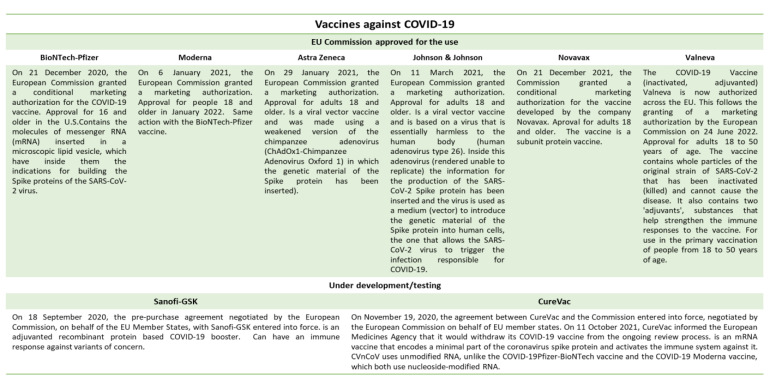
To date, five safe and effective vaccines against COVID-19 have been following positive scientific recommendations. Others are in the process of experimentation [56,57].

Both drug therapy and individual protective devices are needed to fight an infectious viral or bacterial pandemic. As in the current case of the COVID-19 pandemic, therapy cannot always prevent contagion. Current therapy for COVID-19 is based on dexamethasone, tocilizumab, remdesivir, and baricitinib in combination with remdesivir, anticoagulation drugs, three monoclonal antibody treatments (authorized by the FDA), and corticosteroids (such as dexamethasone) [47]. Since the first vaccine conceptualization and development, humans have eradicated some serious infections such as smallpox and polio, but have also sought to prevent other forms of infection. It must be mentioned that in the first quarter of 2020, there was a drop in vaccination coverage from other infections from 10% to 50% compared to 2019 due to the implementation of strict social and physical distancing policies in many countries around the world. Indeed, during this same period, polio, measles, and other vaccination campaigns were suspended due to concern over COVID-19 transmission in campaign settings [48]. The content of the vaccine introduces its component into the body and is recognized by the immune system, subsequently, when the body encounters the foreign entity again, the bacterium is activated and develops antibodies and memory cells. This is thanks to immunological memory, which can last for several years (thus protecting us from possible reinfections) [49]. There are various types of vaccine that differ in their compositions, namely: (a) live attenuated, which contains viruses or bacteria that are still alive but rendered harmless and therefore no longer capable of causing disease (such as that used for measles); (b) inactivated, in which viruses or bacteria have been killed through heat or with a chemical treatment (such as polio or IPV); (c) subunit vaccines (do not contain any whole bacteria or viruses) such as the recombinant protein (hepatitis B, HPV, and meningococcal B or MenB vaccines), toxoid (diphtheria, tetanus, pertussis vaccines), conjugate (H. influenzae b or Hib, meningococcal C or MenC, pneumococcal conjugate or PCV vaccines), virus like particles (hepatitis B and human papilloma virus or HPV vaccines) and the outer membrane vesicles (MenB or meningococcal B vaccine); (d) nucleic acid vaccines are the messenger RNA (such as mRNA COVID-19) and DNA vaccines (currently not licensed); and (e) viral vectored (have a harmless viruses to release the genetic code) and can be replicating (such as the Ebola vaccine) or non-replicating (such as the COVID-19 vaccine) [50,51]. Until a high level of stability is achieved and the quality of microbiology laboratory methods for SARS-Cov-2 detection is maintained, the results of laboratory methods should be evaluated with caution in various clinical situations. Physicians are strongly advised to consult the updated recommendations of the WHO and global organizations on the availability and use of new diagnostic methods. It is possible that the SARS-CoV-2 virus persists in an unrecognized animal reservoir from which it can once again jump into the human population. There are currently effective vaccines in animals to prevent coronavirus infections [7,9]. The effectiveness of the vaccine was first demonstrated in appropriate animal models, and it takes time to demonstrate its safe use in humans. Several factors such as age, comorbidities, and variants of the virus can have a negative impact on the effectiveness of vaccines [51,52]. In fact, the WHO reports that it is not yet known how long the immunity from the various vaccines currently in circulation will last. For this reason, all public health measures including physical distancing, face masks, and hand washing should be used. It has been noted that protection gradually increases and, that after 14 days, significant levels of protection occur. For a single-dose vaccine, protection is generally believed to occur within two weeks of vaccination compared to two-dose vaccines [53]. The vaccine types currently in use for SARS-CoV-2 are as follows: (a) mRNA (messenger RNA, which contains material from the virus that causes infections); (b) protein subunit, which includes harmless pieces (proteins) of the virus that causes COVID-19 instead of the entire germ; and (c) the viral vector, which contains a modified version of a different virus with material from the virus that causes COVID-19 (Figure 5) [54]. The function of the mRNA vaccine is to transmit a message of life contained in the DNA so that the cell can subsequently use it to produce proteins that are needed for life. RNA is a fragile molecule because it is usually present in the cell during its action, as opposed to DNA. For this reason, this type of vaccine can be stored at temperatures as low as −90 °C [54]. The mRNA is encased inside spheres made of liposomes. Once injected into the human body, these lipid spheres release the specific mRNA. In fact, it contains the information to produce the spike structural protein of the virus; this is the key to cells multiplying and causing infection. Once the mRNA vaccine enters the cells, ribosomes translate its information into proteins; in other words, they produce many copies of the SARS-CoV-2 spike protein. Once produced, it leaves the cell and is recognized as a foreign entity by the immune system, which activates a reaction against it but without causing disease; this is because it represents only a small part of the viruses’ structure, but it also triggers the memory cells (Figure 5) [55].

According to the WHO, globally, up to 17 August 2022, we had 589,680,368 confirmed cases, 6,436,519 deaths, reported to WHO, and up to 9 August 2022 a total of 12,355,390,461 vaccine doses were administered [58].

An unprecedented worldwide effort to develop safe and effective COVID-19 vaccines began in January 2020 and rolling out in December 2020. The goal of facilitating fair and equitable access to COVID-19 vaccines gave birth to the institution of the COVAX Global Vaccine Facility co-led by the WHO, Gavi, and the Coalition for Epidemic Preparedness Innovations, in collaboration with UNICEF and others. In addition, the Strategic Advisory Group of Experts on Immunization (SAGE) approved by the WHO gave recommendations for any COVID-19 vaccine [59].

**Figure 6:**
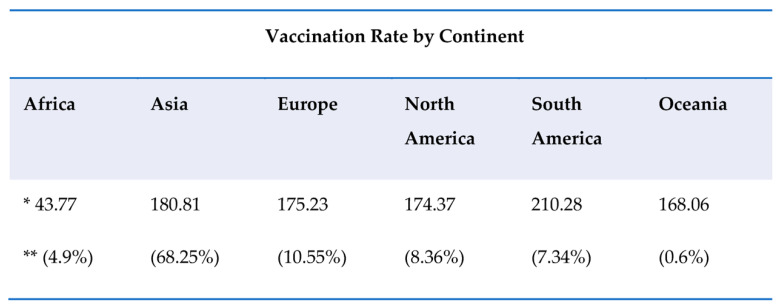
The vaccination trend rate in the continents until 17 August, 2022. The WHO target is 70% of the vaccinated population by mid-2022. According to the WHO, 67.4% of the world’s population has received at least one dose of the COVID-19 vaccine. * Doses administered per 100 inhabitants, ** Share of the world total of doses administered [65,66,67,68].

The involvement of the pharmaceutical–diagnostic industry is clearly desirable and necessary, but the problems that may arise from “exclusive copyright” must not create obstacles in the pursuit of scientific development [55]. According to the European Center for Disease Prevention and Control (ECDC), the assessment of the risk represented by the SARS-CoV-2 pandemic and the predominance of the Delta variant can be evaluated according to the division of the total general population into two groups: the vaccinated and the unvaccinated, which themselves contain two other groups—the vulnerable vaccinated and the vulnerable unvaccinated. This stratification occurs through some important considerations. Vaccinated people are less likely to become infected, and the impact of the disease is less pronounced than it is in unvaccinated people. Moreover, the population that is vulnerable is more likely to suffer from severe infection. In countries with COVID-19 vaccination coverage equal to or below the current EU average level in the total population, and those planning to ease very high viral circulation non-pharmaceutical interventions (NPIs), fully vaccinated vulnerable populations are also at risk of infection, which could lead to a severe outcome [48,60,61,62,63,64]. In contrast, countries with COVID-19 vaccination coverage above the current EU average level, particularly those with the highest current coverage in the total population, have a lower risk in this respect, unless there is a rapid decline in vaccine efficacy due to decreased immunity. In EU countries with COVID-19 vaccination coverage equal to or lower than the current average (60–80%) level in the total population, and those that plan the easing of non-pharmaceutical interventions, viral infections (high viral circulation) will be very high, meaning that vulnerable vaccinated populations are also at risk of infection, which could lead to a severe outcome. Conversely, countries with a vaccination coverage above the current average (60–80%) or high level (80%>) in the total population have a lower risk in this respect, unless there is a rapid decline in vaccine efficacy due to decreased immunity (Figure 6) [64].

Finally, an experimental model found that the highest risk of establishment of resistant strains occurs when a large fraction of the population has already been vaccinated but transmission is not controlled [61,65].

Currently, the goal of the COVID-19 vaccination campaign around the world continues to be essential because it reduces hospitalizations, complications, and deaths while protecting health systems, so vaccination has continued with three booster doses, even extending from the age of 5 years old [66]. According to the WHO, nearly one billion people in low-income countries are not vaccinated. Only 57 countries have vaccinated 70% of their population, almost all of them high-income countries. In fact, the latest mean of doses administered each day (in the seven last days) in low- and middle-income countries is 425,961. At this rate, the target to cover 70% of the vaccinable population would be reached by January 2030 [67,68]. The inequalities related to COVID-19 vaccination access in the population must be effectively addressed. In general, it is crucial to discover the factors that determine low vaccine prevalence (including issues related to vaccine acceptance and access), so that targeted, effective interventions can be carried out to resolve the issue of correct vaccination diffusion in the population. As for future vaccination strategies, they may also differ depending on the availability of updated vaccines and their characteristics. In fact, various nations can use not only one type of vaccine, but different types for different vaccination strategies depending on the characteristics of the development of the vaccines updated, compared to those used before and considering the emergence of new variants [69].

**Figure 7:**
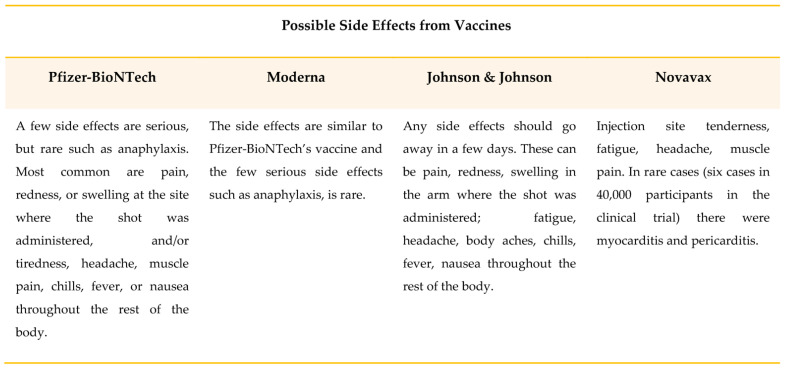
The possible adverse reactions or side effects of the vaccines approved and used to date [70].

Like all other vaccines, COVID-19 vaccines can have mild and short-term side effects. These can include fever, fatigue, headache, body aches, chills, diarrhea, and pain at the site of inoculation. However, rather serious (such as allergic reactions) or possibly long-lasting side effects are possible, but are exceedingly rare [70]. Based on available evidence, the WHO recommends that people with a history of severe allergic reactions to any ingredient in the COVID-19 vaccines should generally not undergo vaccination (Figure 7).

According to the CDCs, vaccination for COVID-19 is recommended for pregnant women or those trying to get pregnant now or who may become pregnant in the future and while breastfeeding. Pregnant women can also be given the booster dose [63,64].

Furthermore, the evidence on the safety and efficacy of vaccination during pregnancy is increasing. The WHO, on 2 June 2021, recommended vaccination in pregnant women when the benefits of vaccination for the pregnant woman under potential risks (e.g., women who are at high risk of exposure to COVID-19 during pregnancy or with comorbidities that place them in a high-risk group for severe COVID-19). Furthermore, the WHO recommends vaccination in lactating women as in other adults. However, scientific authorities such as the CDC, the WHO, and regulatory authorities monitor the use of vaccines for COVID-19 to identify any problems regarding their safety that could arise to ensure their safe use throughout the global population [64].

Finally, it must be mentioned that the adaptive natural immune response is very important for defense, and thus for patient outcome after SARS-CoV-2 infection and supports the efficacy of the vaccine. The T-cell responses develop early and are related to protection. The T-cell memory includes extensive recognition of viral proteins, estimated at approximately 30 epitopes within everyone (in fact, current vaccines have focused on the spike protein, which contains the ligand epitope for the ACE2 receptor). However, this natural immune memory could limit individual viral mutations and is likely to support protection against severe disease from COVID 19 viral variants (such as Omicron) [71,72]. Hence, the whole COVID-19 (not just a part) provides a wider variety of peptides than antigenic surfaces that T cells can recognize. Current COVID-19 vaccines facilitate potent responses of adaptive immune, and thus the T cells. These defense cells likely contribute to substantial protection against hospitalization or even avoidance of fatality, and novel or heterologous regimens offer the potential to improve further cellular responses. Hence, the immunity that T lymphocytes offer plays a crucial role in the control of the viral disease through the elimination of infected cells, and its importance may have so far been relatively underestimated. On the other hand, the prevention of infection by the host in general or by individual cells will be the responsibility of B-lymphocytes. Therefore, although natural adaptive immunity is better “trained”, that is, more prepared in its surveillance through T-lymphocytes, to give a more robust and effective response, than that induced by vaccines with only the “S” (spike) protein [7,72].

In a retrospective observational study involving 124,500 persons, they were divided into two groups and compared to each other. The first was with SARS-CoV-2 naïve individuals who received a two-dose regimen of the BioNTech/Pfizer mRNA BNT162b2 vaccine, and the second was previously infected individuals who were never vaccinated, acquired immunity of the infected, but unvaccinated people, confers stronger protection against infection with the Delta variant of SARS-CoV-2 than the two-dose vaccine-induced immunity of BioNTech/Pfizer mRNA BNT162b2 [73]. Additionally in another observational cohort study, they evaluated the antibody and cellular immune responses following COVID-19 vaccinations in members of staff and residents at 74 long-term care facilities (LTCFs) across the UK. It was found that the suboptimal post-vaccine immune responses in LTCF-naïve residents needed to improve immune protection through a second dose of vaccine [74].

## 5. Conclusions

The evolution of microorganisms (i.e., the process that leads to change that allows them to adapt to the environment) is a consequence of genotypic modification. This process occurs when there is less genomic complexity, as shown in the evidence. For this reason, in the case of viruses, and given the reduced number of genes they possess, they have the fastest evolution; this is the main success factor that allows them to adapt and survive. Among the viruses, RNA viruses show extreme variability and are responsible for pandemics, as has been noted in recent years. Coronaviruses, therefore, show a considerable propensity to transmit themselves to new hosts. Thus, there are several challenges facing the field of microbiology in the future including those posed by SARS-CoV-2. It has been noted that a key challenge is between prevention and rapid recognition, not only of the agent, but also of its virulence. One of the main problems is how we might conduct the effective surveillance of variants and how these variants might affect virus transmissibility, diagnostics, therapy, vaccine efficacy, and effective antivirals as well as how effective reinfection surveillance might be conducted in relation to SARS-CoV-2. Although it is not easy to predict exactly how infectiousness, pathogenicity, and the possibility of the virus “escaping” from the immune system might play out over time, we can predict what factors could influence these trends. One parameter is the immunity that has already built up the population that reduces the possibility of transmission and limits the development of new mutations.

Finally, in the context of a pandemic and the continuous spread of an infectious agent, there must be an increase in the capacity for control at an international level and the collection of evidence on the elimination of the virus and its infectivity, it is necessary to update the present guidelines aimed at that infectious agent to put an end to the total isolation of communities or groups or individuals. Guidelines must then reflect on the information available at the time of publication and may change depending on the current epidemiological and scientific data of the pandemic. An early recognition by the health institutions in the possible pandemic zone, and relying on an R0, redeems the response faster. The exact duration of infectivity of patients must be known with certainty. For example, the greatest risk of transmission occurs in the period close to the onset of symptoms and may be initially detected in secretions such as those of the upper respiratory tract, possibly earlier than the appearance of symptoms. In our era, travel between cities and continents is easier than in the past, and this makes it necessary to control travelers first. Therefore, the crucial points of the management of a pandemic can be summarized as follows: (a) epidemiological surveillance measures and procedures by means of case definition, diagnostic confirmation procedures, tracing of contacts, telemedicine and psychological support; (b) management attention of some subgroups with special needs (such as pregnancy, pediatric age, patients with comorbidities and other); (c) prevention and containment measures (prevention measures for the entire population, prevention measures for health workers, personal protective equipment (PPE) for health workers); (d) collaboration between the international public health strategies (such as WHO, CDCs USA, and ECDC and others) with the various National Institutions; and (e) must remember the importance of correct communication between the institutions, health workers, and population.

We believe that our current pandemic has taught us that we are too close in our remoteness.

## Data Availability

Data are contained within the article.
